# Inhibition of DNA Repair in Combination with Temozolomide or Dianhydrogalactiol Overcomes Temozolomide-Resistant Glioma Cells

**DOI:** 10.3390/cancers13112570

**Published:** 2021-05-24

**Authors:** Shigeo Ohba, Kei Yamashiro, Yuichi Hirose

**Affiliations:** Department of Neurosurgery, Fujita Health University, Toyoake 4701192, Japan; ykei@fujita-hu.ac.jp (K.Y.); yhirose@fujita-hu.ac.jp (Y.H.)

**Keywords:** dianhydrogalactiol, drug resistance, glioma, PAPR inhibitor, temozolomide

## Abstract

**Simple Summary:**

Glioblastoma is the most prevalent and lethal brain tumor. Temozolomide is usually used for the treatment of glioblastoma. The poor prognosis of the tumor is due to drug resistance and tumor heterogeneity. The mechanism of the resistance to temozolomide is various within the same tumor. The aim of the study was to clarify the mechanism of temozolomide resistance and find methods to overcome temozolomide resistance in glioma. Inhibition of DNA repair (homologous recombination or base excision repair) resensitized resistant cells harboring different resistance mechanism to temozolomide. Additionally, a bifunctional DNA-targeting agent, dianhydrogalactiol, showed anti-tumor effect independent of MGMT and mismatch repair status. Further, inhibition of checkpoint or homologous recombination enhanced dianhydrogalactiol-induced cytotoxicity in temozolomide-resistant glioma cells. Although resistance to temozolomide is clinically important issue, selecting suitable treatments for resistance mechanism can improve the prognosis of glioma.

**Abstract:**

Resistance to temozolomide and intratumoral heterogeneity contribute to the poor prognosis of glioma. The mechanisms of temozolomide resistance can vary within a heterogeneous tumor. Temozolomide adds a methyl group to DNA. The primary cytotoxic lesion, O6-methylguanine, mispairs with thymine, leading to a futile DNA mismatch repair cycle, formation of double-strand breaks, and eventual cell death when O6-methylguanine DNA methyltransferase (MGMT) is absent. N7-methylguanine and N3-methyladenine are repaired by base excision repair (BER). The study aim was to elucidate temozolomide resistance mechanisms and identify methods to overcome temozolomide resistance in glioma. Several temozolomide-resistant clones were analyzed. Increased homologous recombination and mismatch repair system deficiencies contributed to temozolomide resistance. Inhibition of homologous recombination resensitized resistant cells with high homologous recombination efficiency. For the mismatch repair-deficient cells, inhibition of BER by PARP inhibitor potentiated temozolomide-induced cytotoxicity. Dianhydrogalactiol is a bifunctional DNA-targeting agent that forms N7-alkylguanine and inter-strand DNA crosslinks. Dianhydrogalactiol reduced the proliferation of cells independent of MGMT and mismatch repair, inducing DNA double-strand breaks and apoptosis in temozolomide-resistant cells. Further, inhibition of chk1 or homologous recombination enhanced dianhydrogalactiol-induced cytotoxicity in the cells. Selecting treatments most appropriate to the types of resistance mechanisms can potentially improve the prognosis of glioma.

## 1. Introduction

Glioblastoma is one of the most aggressive brain tumors, with a median survival of about 1.5 years [1]. The standard treatment for glioblastomas is maximal safety resection, followed by radiation therapy and temozolomide (TMZ) [2]. TMZ is an alkylating agent that adds a methyl group to DNA. TMZ-induced DNA methylation of 5%, 60% to 70%, and 10% to 20% occur at the O6 position of guanine, N7 position of guanine, and N3 position of adenine, respectively [3]. O6 methylguanine is repaired by O6-methylguanine DNA methyltransferase (MGMT), whereas N7 methylguanine and N3 methyladenine are repaired by base excision repair (BER) [4]. TMZ-induced cytotoxicity is mainly derived from O6-methylguanine. O6-methylguanine mispairs with thymine, and this mispair is repaired by the mismatch repair (MMR) system. The futile cycles of MMR have been shown to induce DNA double-strand breaks (DSBs) and eventually cell death [5].

TMZ-induced N7-methylguanine and N3-methyladenine are sensed by the BER pathway [6]. DNA glycosylases recognize and excise damaged bases and initiate the repair process. The poly (ADP-ribose) polymerase (PARP) family of enzymes coordinates DNA damage responses and binding to strand breaks in DNA. After binding, PARP becomes catalytically activated and synthesizes PAR polymers attached to itself and other repair factors. The BER complex is recruited more efficiently to sites of DNA damage [7,8].

Dianhydrogalactiol (DAG) is a bifunctional DNA-targeting agent forming N7-alkylguanine and inter-strand DNA crosslinks [9]. DAG is a small water-soluble molecule that can cross the blood–brain barrier (BBB) [10]. DAG has been approved for the treatment of chronic myeloid leukemia and lung cancer in China [11].

Because there have been no additional effective treatment for recurrent tumors after treatment of TMZ, it is very important to investigate potential methods to overcome resistance in gliomas. On the other hand, because glioblastoma is a heterogeneous tumor, the mechanisms of resistance to TMZ are considered to be different. The study aim was to investigate the mechanisms of resistance to TMZ by using clones of resistant glioma cells, and find the appropriate treatment for each resistant cell.

## 2. Materials and Methods

### 2.1. Cell Culture, Creation of TMZ-Resistant Cells, and Reagents

U251, U87, and SF767 cells were cultured in Dulbecco’s modified Eagle’s medium supplemented with 10% fetal bovine serum and 1% penicillin/streptomycin at 37 °C in a 5% CO_2_ atmosphere. TMZ-resistant clones (U251, U87) were derived from the TMZ-sensitive U251 or U87 cells by culturing them with increasing doses of TMZ, as described previously [12].

Cells were treated with each reagent: TMZ (LKT Laboratories, St. Paul, MN, USA), VAL-083 (MedChemExpress, Shanghai, China), talazoparib, veliparib, MK-8776 (Selleck Chemicals, Houston, TX, USA), and Olaparib (ChemScene, Monmouth Junction, NJ, USA).

### 2.2. Genetic Suppression of RAD51, or MSH6

U251-derived TMZ-resistant cells were plated at 10^5^/mL in 6-well plates in DMEM media without antibiotics. Twenty-four hours later, the cells were transfected with an optimized amount (5 nmol/L) of siRNA targeting human RAD51 (Dharmacon, Lafayette, CO, USA) or non-targeting siRNAs using DharmaFECT reagent (Dharmacon) according to the manufacturer’s protocol as described previously [13].

### 2.3. Protein Extraction and Immunoblot Analyses

Cells were lysed in RIPA Lysis and Extraction Buffer (Thermo Fisher Scientific, Waltham, MA, USA) supplemented with 1× PhosStop and protease inhibitor cocktail (Roche, Basel, Switzerland). Protein (30 μg) was used for western blot analysis using each primary antibody and appropriate horseradish peroxidase-conjugated secondary antibody.

### 2.4. Cell Cycle Studies

At each time point, attached and floating cells were collected, fixed, and stained with PI (Sigma-Aldrich, St. Louis, MO, USA) as described [14]. Cells were subjected to flow cytometry and analysis using a Gallios (Beckman Coulter Life Sciences, Brea, CA, USA) and Kaluza analysis software version 2.1 (Beckman Coulter Life Sciences, Brea, CA, USA).

### 2.5. Immunofluorescence Studies

For immunofluorescence studies, cells were seeded onto four-well glass coverslips, incubated with TMZ and/or talazoparib or MK-8776 and/or VAL-083, washed in phosphate-buffered saline (PBS), fixed with 4% paraformaldehyde in PBS (15 min, room temperature), rinsed with PBS, and blocked in PBS containing 0.1% Triton-X and 10% FBS (1 h, room temperature). The cells were stained with anti-Ser-139-phosphorylated H2AX antibody (Cell Signaling Technology, Danvers, MA, USA) at 1:1000 dilution or anti RAD51 antibody at 1:500 dilution followed by Alexa 488 (Cell Signaling Technology) or Alexa 594 (Thermo Fisher Scientific, Waltham, MA, USA) conjugated secondary antibody (30 min, room temperature). Cells were washed, counterstained with 4′,6′ diamidino-2-phenylindole, and mounted with fluorescence mounting medium (Dako, Santa Clara, CA, USA). Quantitative analysis was performed by using the number of cells with nuclei containing >5 γ-H2AX or RAD51 foci determined by fluorescence microscopy [13].

### 2.6. Colony Formation Efficiency

The colony formation efficiencies of the control and drug-treated cells were determined by performing a colony formation assay, as previously described [14]. Briefly, the cells were plated at a concentration of 100 cells/well into 6-well culture plates 2 days prior to drug treatment. After incubation with each drug, the cells were incubated in a drug-free medium and allowed to form colonies. The cells were stained with methylene blue (Sigma-Aldrich, St. Louis, MO, USA), and colonies with >50 cells were counted 14 days after drug exposure.

### 2.7. Measurement of HR Efficiency

An HR efficiency assay was performed by using a quantitative polymerase chain reaction-based HR assay kit (Norgen Biotek Corp, Thorold, ON, Canada), as described previously [13]. Briefly, the system contains two plasmids with a different mutation in its lacZ coding region. After cotransfection, the total cellular DNA was isolated. A set of universal primers that amplify all plasmid DNA or a set of primers that only amplify plasmid DNA generated by HR of the transfected plasmids were used in qPCR reactions. The amount of recombinant product for each reaction was calculated by comparing the cycle number at the point of inflection of the amplification curve generated using the HR-specific primers to that using universal primers, and converting the difference in cycle number to a DNA amount by comparison with a standard curve generated using universal primers and different amounts of input DNA.

### 2.8. Statistical Analyses

Data are reported as the mean ± standard error of at least three experiments. When two groups were compared, the unpaired Student’s t-test was applied. When multiple groups were evaluated, the one-way ANOVA test with post hoc Turkey–Kramer multiple comparisons test was used, or Dunnett’s test was used, comparing to the value of untreated cells. Values of *p* < 0.05 were considered to be indicative of statistical significance.

## 3. Results

This section may be divided by subheadings. It should provide a concise and precise description of the experimental results, their interpretation, as well as the experimental conclusions that can be drawn.

### 3.1. TMZ-Resistant U251 Clones Showed Different Response to TMZ

**Figure 1:**
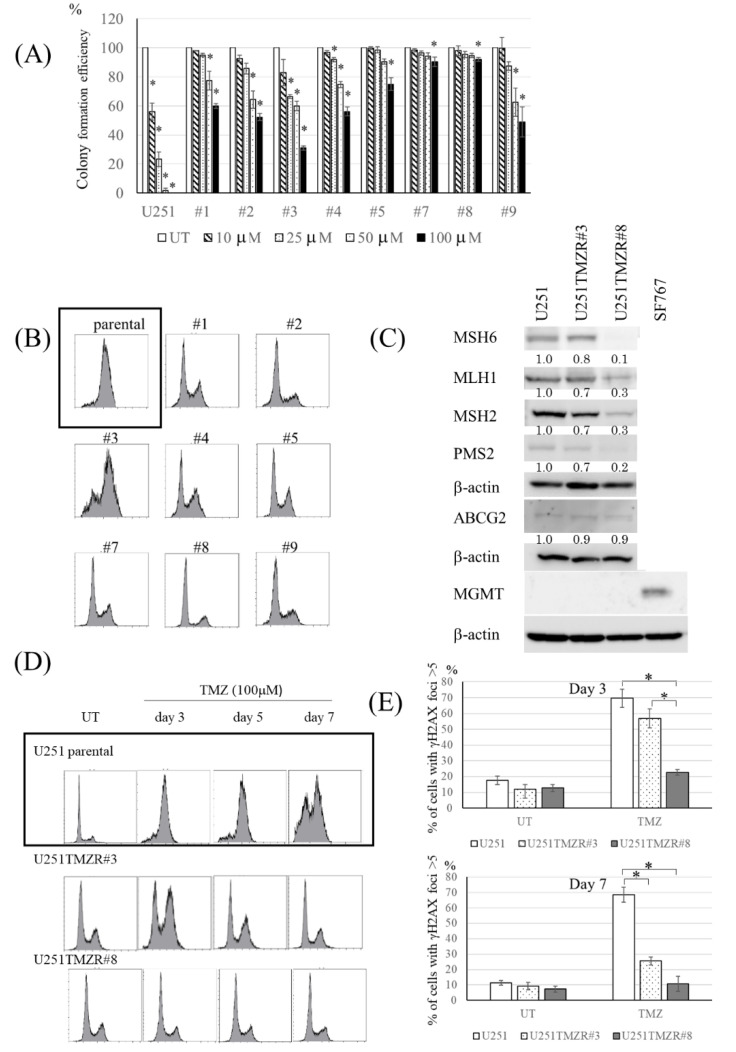
TMZ-resistant U251 clones had different characters. (**A**) Colony formation efficiency of U251 and U251-derived TMZ-resistant clones following TMZ exposure (0–100 μM, 3 h). (**B**) Fluorescence-activated cell sorting (FACS) analysis of cell cycle distribution in U251 (parental) and U251-derived TMZ-resistant clones 3 days after TMZ (100 μM, 3 h) exposure. Each clone showed different patterns of cell cycle phase distributions. (**C**) Western blot analysis of MMR-related proteins (MLH1, MS2H, MSH6, and PMS2), ABCG2, MGMT, and β-actin levels in U251 parental, U251TMZR#3, and U251TMZR#8 cells. SF767 cells were used as positive control for MGMT expression. (**D**) FACS analysis of cell cycle distribution in U251 (parental), U251TMZR#3, and U251TMZR#8 cells 3–7 days after TMZ (100 μM, 3 h) exposure. TMZ-induced G2/M arrest rmained at least by day 7 in U251 (parental) cells. TMZ-induced G2/M arrest recovered sooner in U251TMZR#3, whereas TMZ did not induce G2/M arrest in U251TMZR#8. (**E**) Quantitative data from immunofluorescence analysis of γ-H2AX foci in U251, U251TMZR#3, and U251TMZR#8 cells 3 and 7 days after TMZ exposure (100 μM, 3 h)., * *p* < 0.05.

To start the experiments, the characteristics of TMZ-resistant U251 clones were evaluated. The colony formation efficiency assay revealed that each clone showed resistance to TMZ (Figure 1A). After exposure to TMZ, different patterns of cell cycle phase distributions were observed (Figure 1B). Because several mechanisms are associated with resistance to TMZ, such as MMR deficiency and high expression of MGMT [15,16], the expression of MMR-associated proteins and MGMT were measured. The clones showed different patterns of MLH1, MSH2, MSH6, and PMS2 expression, but did not show the expression of MGMT (Figure 1C, Figure S1).

TMZ-resistant U251 #3 (U251TMZR#3) clone, which showed the largest G2/M population, and TMZ-resistant U251 #8 (U251TMZR#8) clone, which showed the smallest G2/M population (Figure 1B), were used in further experiments. Expression of MSH6 was not suppressed in #3 clone but was in #8 clone (Figure 1C). In U251TMZR#3 clone TMZ-induced G2/M arrest recovered sooner than in parental cells, whereas TMZ did not induce G2/M arrest in #8 clone (Figure 1D). Consistent with these findings, TMZ-induced γ-H2AX foci disappeared more rapidly in U251TMZR#3 clone than in parental cells, and the foci did not show as great of a change between with and without TMZ in U251TMZR#8 (Figure 1E).

### 3.2. Inhibition of HR Resensitized U251TMZR#3 Clone, But Not #8 Clone, to TMZ

**Figure 2:**
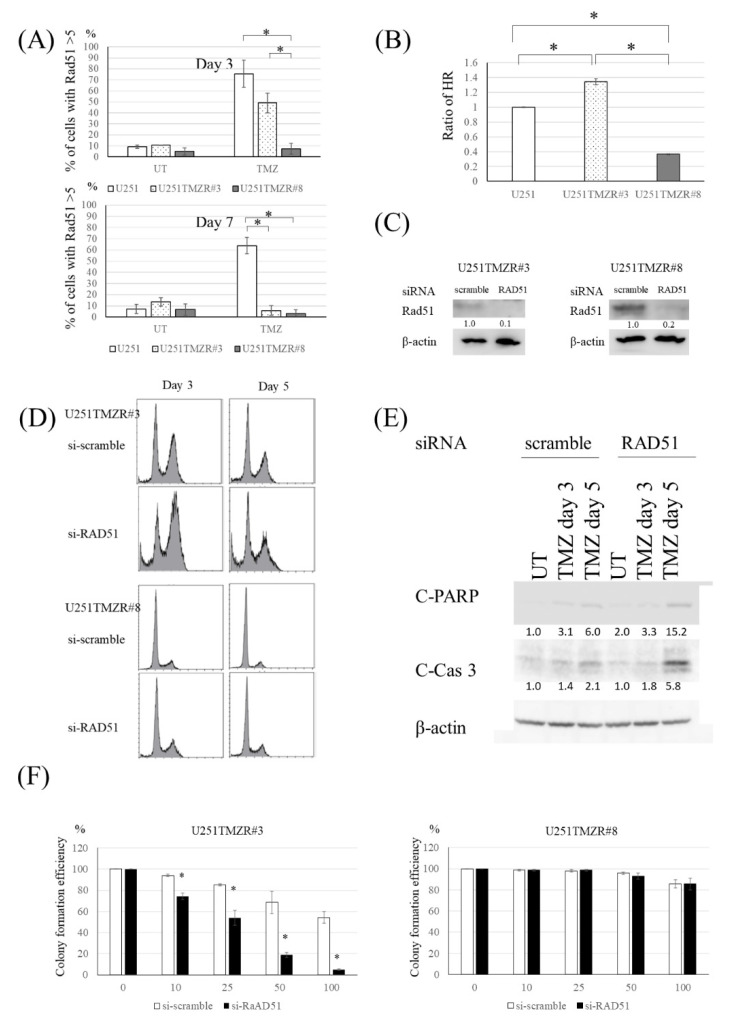
Inhibition of HR resensitized the TMZ-resistant U251 cells harboring high HR to TMZ. (**A**) Quantitative data from immunofluorescence analysis of Rad51 foci in U251, U251TMZR#3, and U251TMZR#8 cells 3 and 7 days after TMZ exposure (100 μM, 3 h). (**B**) Homologous recombination activity as determined by an in vivo plasmid-based recombination reporter assay. Value of the U251 cells was set at 1. (**C**) Western blot analysis of RAD51 and β-actin levels in U251TMZR#3 and U251TMZR#8 cells three days following exposure to scramble or RAD51-targeting siRNA. (**D**) Fluorescence-activated cell sorting analysis of cell cycle distribution in control and RAD51-suppressed U251TMZR#3 and #8 cells 3 and 5 days after TMZ exposure (100 μM, 3 h). (**E**) Western blot analysis of cleaved PARP, C-leaved caspase 3, and β-actin levels in U251 parental, U251TMZR#3, and U251TMZR#8 cells. (**F**) Colony formation efficiency of control and RAD51-suppressed U251TMZR#3 and #8 cells following TMZ exposure (0–100 μM, 3 h)., * *p* < 0.05.

TMZ-induced DNA DSBs are repaired in human cells by the HR process [17,18]. HR requires a variety of proteins, including Rad51 accumulating at the sites of DSBs [13]. Parental and resistant U251 cells exhibited a similar pattern of Rad51 foci after TMZ exposure to the results of γ-H2AX foci (Figure 2A), suggesting that an HR-related event was activated in U251TMZR#3 clone. We used a plasmid recombination-based method [13] to determine whether HR was increased in #3 clone. Compared with parental cells, U251TMZR#3 clone showed higher HR ability (Figure 2B). To confirm that the increased HR was responsible for the resistance to TMZ in the #3 clone, further studies using a genetic inhibitor of RAD51 were performed. Introduction of RAD51 siRNA inhibited the ability of the #3 clone to shorten TMZ-induced G2/M arrest, induced apoptosis, and resensitized U251TMZR#3 clone to TMZ (Figure 2C–F).

### 3.3. PARP Inhibitor Resensitized U251TMZR#8 Clone to TMZ

**Figure 3:**
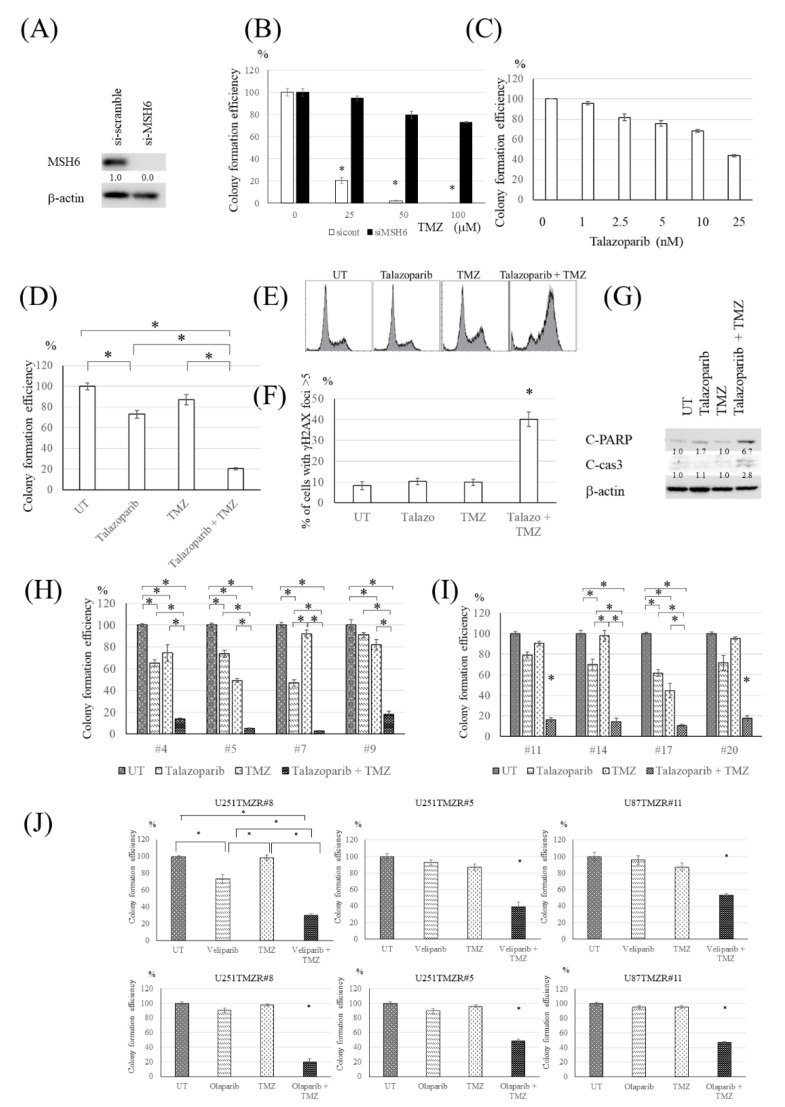
PARP inhibitor resensitized the TMZ-resistant U251 cells harboring MMR deficiency to TMZ. (**A**) Western blot analysis of MSH6 and β-actin levels in U251 cells treated with siRNA targeting MSH6 or non-targeting siRNA. (**B**) Colony formation efficiency of control and MSH6-suppressed U251 cells following TMZ exposure (0–100 μM, 3 h). (**C**) Colony formation efficiency of U251TMZR#8 cells following talazoparib exposure (0–25 nM, 4 days). (**D**) Colony formation efficiency of U251TMZR#8 cells following talazoparib (2.5 nM, 4 days), and/or TMZ (100 μM, 3 h). (**E**) Fluorescence-activated cell sorting (FACS) analysis of cell cycle distribution in U251TMZR#8 after talazoparib and/or TMZ exposure. (**F**) Quantitative data from immunofluorescence analysis of γ-H2AX foci in U251TMZR#8 cells treated with talazoparib and/or TMZ. (**G**) Western blot analysis of cleaved PARP, cleaved caspase 3, and β-actin levels in U251TMZR#8 cells treated with talazoparib and/or TMZ. (**H**) Colony formation efficiencies of U251TMZR#4, #5, #7, and #9 cells following talazoparib (2.5 nM, 4 days) and/or TMZ (100 μM, 3 h). (**I**) Colony formation efficiency of U87TMZR#11, #14, #17, and #20 cells following talazoparib (2.5 nM, 4 days) and/or TMZ (100 μM, 3 h). (**J**) Colony formation efficiency of U251TMZR#8, #5, and U87TMZR#11 cells following veliparib (1 μM, 4 days) or olaparib (1 μM, 4 days) and/or TMZ (100 μM, 3 h)., * *p* < 0.05.

Because MMR-related proteins were suppressed in U251TMZR#8 clone (Figure 1C), the ability of MMR was considered to be inhibited. To confirm that loss of MSH6 was associated with TMZ-resistance, U251 cells were treated with siRNA targeting MSH6. Introduction of MSH6 siRNA inhibited TMZ-induced cytotoxicity (Figure 3A,B). Because MMR was abolished in U251TMZR#8 clone, the effect of the TMZ-induced O6 methylguanine-associated pathway was not expected. Therefore, we investigated another pathway via BER. Talazoparib, a PARP inhibitor, suppressed the clonogenicity of U251TMZR#8 clone in a dose-dependent manner (Figure 3C). Talazoparib resensitized the clone to TMZ at a low concentration that had a lesser effect on colony formation efficiency with talazoparib alone (Figure 3D). The combination treatment of talazoparib and TMZ induced G2/M arrest, DNA DSBs, and apoptosis in the clone (Figure 3E–G). To check that the effect of talazoparib on TMZ-induced cytotoxicity was not specific to the clone #8, other U251-derived resistant clones with suppressed expression of MMR-related proteins were used in the same experiments. The synergistic effect of talazoparib and TMZ was demonstrated in these clones (Figure 3H). The effects of talazoparib on cell cycle distribution and on expression of apoptosis-associated proteins were similar to those on #8 clone (Figure S2A,B). To confirm that the effect was not specific to resistant clones derived from U251, other resistant clones from U87 were used. These resistant clones showed suppressed MSH6 expression and no expression of MGMT (Figure S3A). Talazoparib resensitized TMZ-resistant U87 clones to TMZ, too (Figure 3I). The combination treatment of talazoparib and TMZ induced G2/M arrest and apoptosis in U87-derived resistant cells (Figure S3B,C). To determine if this resensitization was specific to talazoparib or was due to inhibition of PARP, other PARP inhibitors, veliparib or olaparib was used. The synergistic effect of TMZ and veliparib or olaparib was also observed, showing that inhibition of PARP contributed to the effect (Figure 3J).

### 3.4. PARP Inhibitor Resensitized Cells with High Expression of MGMT to TMZ

**Figure 4:**
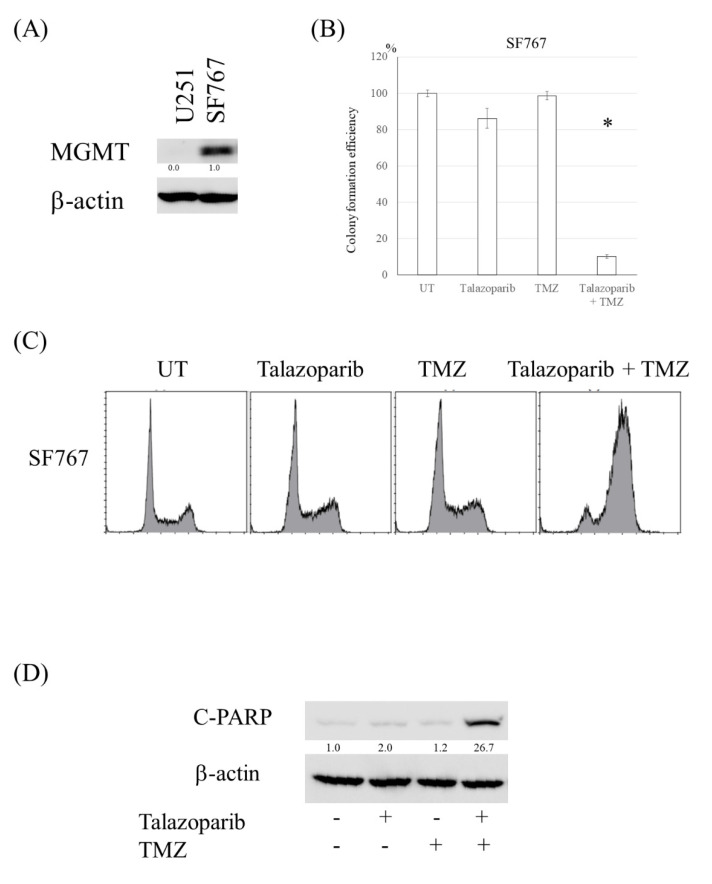
PARP inhibitor resensitized cells with high expression of MGMT to TMZ. (**A**) Western blot analysis of MGMT and β-actin levels in SF767 cells. U251 was used as negative control for MGMT expression. (**B**) Colony formation efficiency of SF767 cells following talazoparib (2.5 nM, 4 days), and/or TMZ (100 μM, 3 h). (**C**) Fluorescence-activated cell sorting analysis of cell cycle distribution in SF767 cells after talazoparib and/or TMZ exposure. (**D**) Western blot analysis of cleaved PARP and β-actin levels in SF767 cells treated with talazoparib and/or TMZ. *, *p* < 0.05.

Because the TMZ-induced methyl group at the O6 position of guanine is removed by MGMT, cells with high expression of MGMT are resistant to TMZ, although the TMZ-resistant clones made in this study did not show the increased expression of MGMT. To confirm that the effect of PARP inhibitor on TMZ in cells with high MGMT expression, SF767 cells were treated with TMZ and talazoparib. The combination treatment reduced colony formation efficiency, increased the G2 population, and induced apoptosis in SF767 cells (Figure 4A–D).

### 3.5. DAG Induced Cytotoxicity in TMZ-Resistant Glioma Cells Independent of MMR Deficiency or MGMT Expression

**Figure 5:**
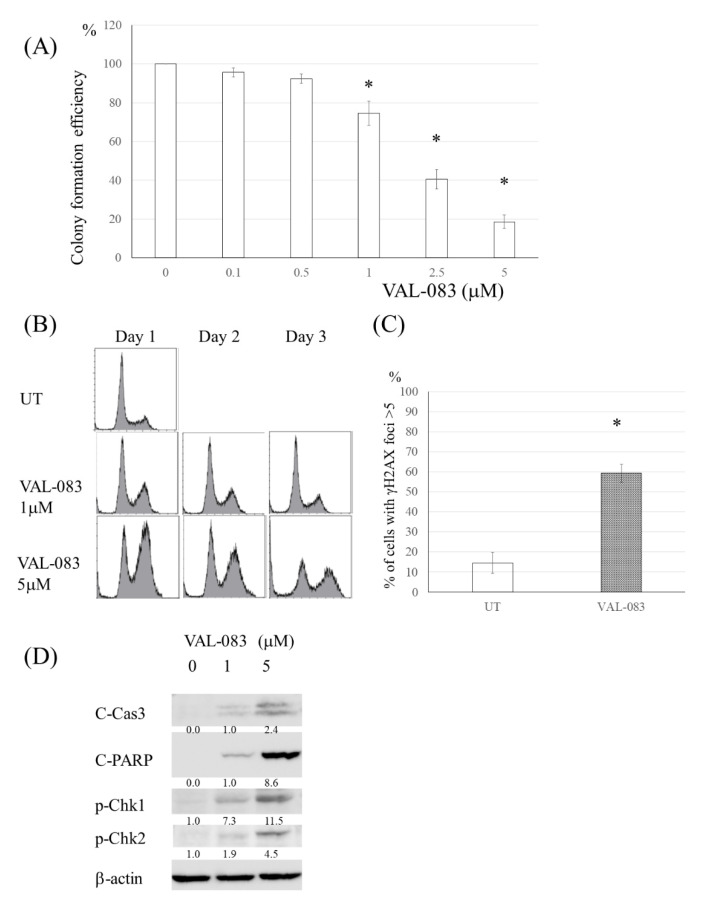
DAG induced cytotoxicity in TMZ-resistant glioma cells independent of MMR deficiency or MGMT expression. (**A**) Colony formation efficiency of U251TMZR#8 cells following VAL-083 exposure (0–5 μM, 3 days). (**B**) Fluorescence-activated cell sorting analysis of cell cycle distribution in U251TMZR#8 after VAL-083 exposure. (**C**) Quantitative data from immunofluorescence analysis of γ-H2AX foci in U251TMZR#8 cells treated with VAL-083. (**D**) Western blot analysis of cleaved PARP, cleaved caspase 3, phosphor-chk1, phosphor-chk2, and β-actin levels inU251TMZR#8 cells treated with VAL-083. *, *p* < 0.05.

Other drugs having different mechanisms of cytotoxicity from TMZ-induced cytotoxicity were expected to be effective in TMZ-resistant glioma cells. The clinical-grade DAG analog VAL-083 reduced the proliferation of U251TMZR#8, increased the G2/M population and induced DNA DSBs (Figure 5A–C). Western blot revealed that VAL-083 activated *p*-chk1 and induced apoptosis (Figure 5D). Because these results were also found in other TMZ-resistant U251, U87 clones, or glioma cells with MGMT expression, it is possible that the effect of VAL-083 was independent of the status of p53, MMR, or MGMT (Figure S4A–C).

### 3.6. Chk1 Inhibitor Enhanced the Cytotoxicity Induced by DAG in TMZ-Resistant Glioma Cells

**Figure 6:**
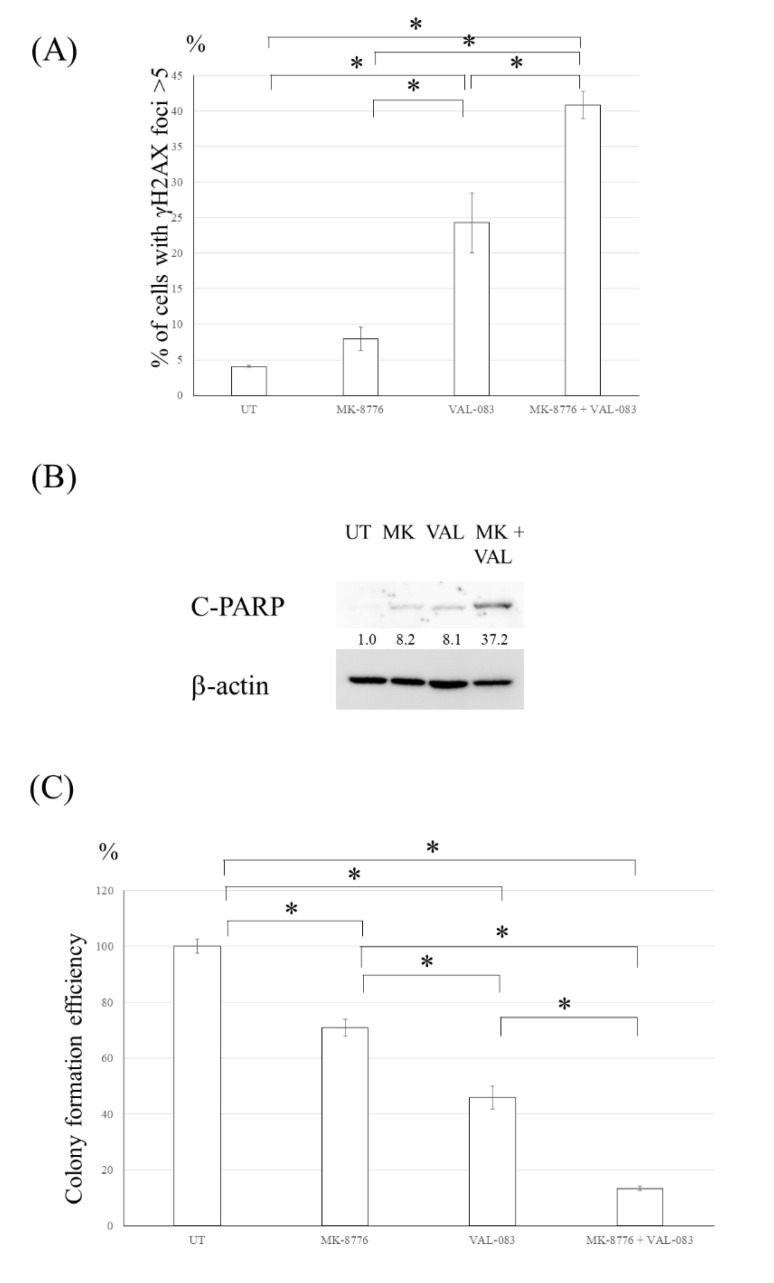
Inhibition of Chk1 enhanced the cytotoxicity induced by DAG in TMZ- resistant glioma cells. (**A**) Quantitative data from immunofluorescence analysis of γ-H2AX foci in U251TMZR#8 cells treated with VAL-083 and/or MK-8776. (**B**) Western blot analysis of cleaved PARP, cleaved caspase 3, and β-actin levels in U251TMZR#8 cells treated with VAL-083 and/or MK-8776. (**C**) Colony formation efficiency of U251TMZR#8 cells following VAL-083 and/or MK-8776 exposure. *, *p* < 0.05.

Because the expression of p-chk1 was increased in the U251TMZR#8 clone treated with VAL-083 (Figure 5D), the effect of a chk1 inhibitor on the VAL-08-induced cytotoxicity was evaluated. A chk1 inhibitor, MK-8776, increased γ-H2AX foci and induced apoptosis in the U251TMZR#8 clone treated with VAL-083 (Figure 6A,B). A colony formation assay revealed that MK-8776 enhanced the cytotoxicity effect of VAL-083 on the U251TMZR#8 clone (Figure 6C).

### 3.7. Inhibition of HR Enhanced the Cytotoxicity Induced by DAG in TMZ-Resistant Glioma Cells

**Figure 7:**
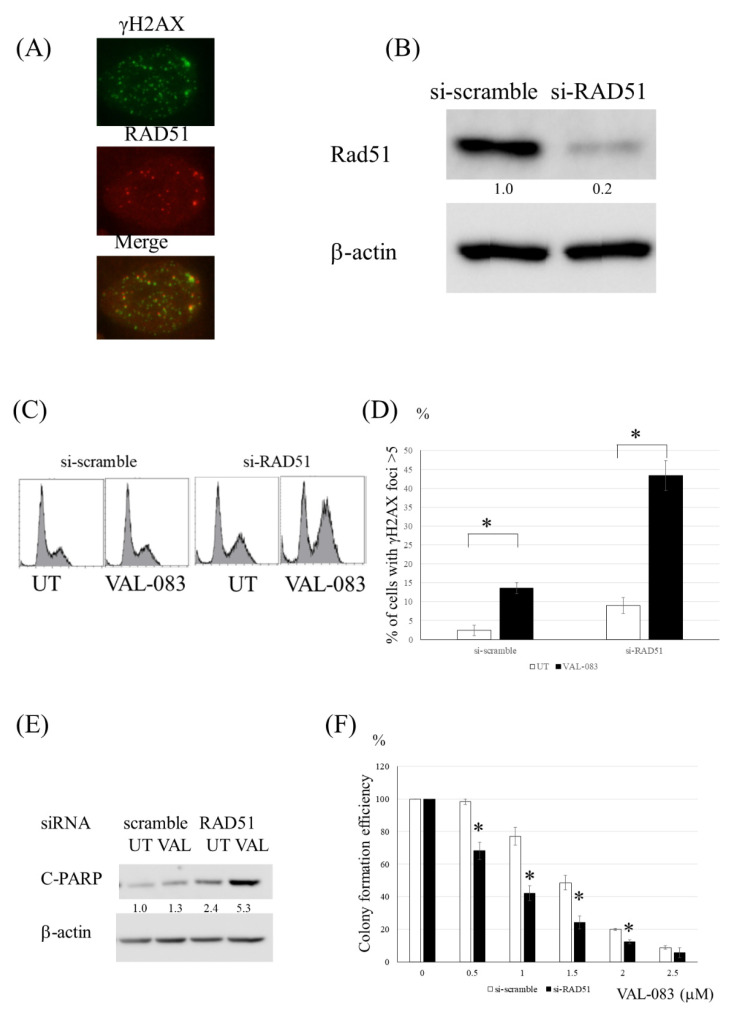
Inhibition of HR enhanced the cytotoxicity induced by DAG in TMZ-resistant glioma cells. (**A**) Immunofluorescence analysis of the physical coincidence of γ-H2AX and RAD51 foci in U251TMZR#8 cells treated with VAL-083. (**B**) Western blot analysis of RAD51 and β-actin levels in U251TMZR#8 cells following exposure to scramble or RAD51-targeting siRNA. (**C**) Fluorescence-activated cell sorting (FACS) analysis of cell cycle distribution in control and RAD51-suppressed U251TMZR #8 cells 2 days after VAL-083 (1 μM) exposure. (**D**) Quantitative data from immunofluorescence analysis of γ-H2AX foci in control and RAD51-suppressed U251TMZR#8 cells treated with VAL-083. (**E**) Western blot analysis of cleaved PARP, cleaved caspase 3, and β-actin levels in control and RAD51-suppressed U251TMZR#8 cells treated with VAL-083. (**F**) Colony formation efficiency of control and RAD51-suppressed U251TMZR#8 cells studied following VAL-083 exposure (0–2.5 μM, 4 days)., * *p* < 0.05.

A fluorescence immunoassay using Rad51 and γ-H2AX revealed that VAL-083 increased the Rad51 and γ-H2AX foci, and these foci were partially colocalized (Figure 7A). These data suggested that HR was associated with the repair of VAL-083-induced DNA DSBs. Therefore, to determine if the inhibition of HR affected the cytotoxicity induced by VAL-083, further experiments using siRNA for Rad51 were performed. The cells treated with VAL-083 and siRNA for Rad51 showed increased G2/M population cells, γ-H2AX foci, and apoptotic cells relative to the cells treated with VAL-083 and non-targeted siRNA (Figure 7B–E). Compared with the introduction of non-targeted siRNA, introduction of RAD51 siRNA enhanced the cytotoxicity of VAL-083 in the U251TMZR#8 clone (Figure 7F). These results revealed that VAL-083-induced DNA DSB was repaired by HR, and inhibition of HR enhanced the VAL-083-induced cytotoxicity in TMZ-resistant glioma cells.

## 4. Discussion

**Figure 8:**
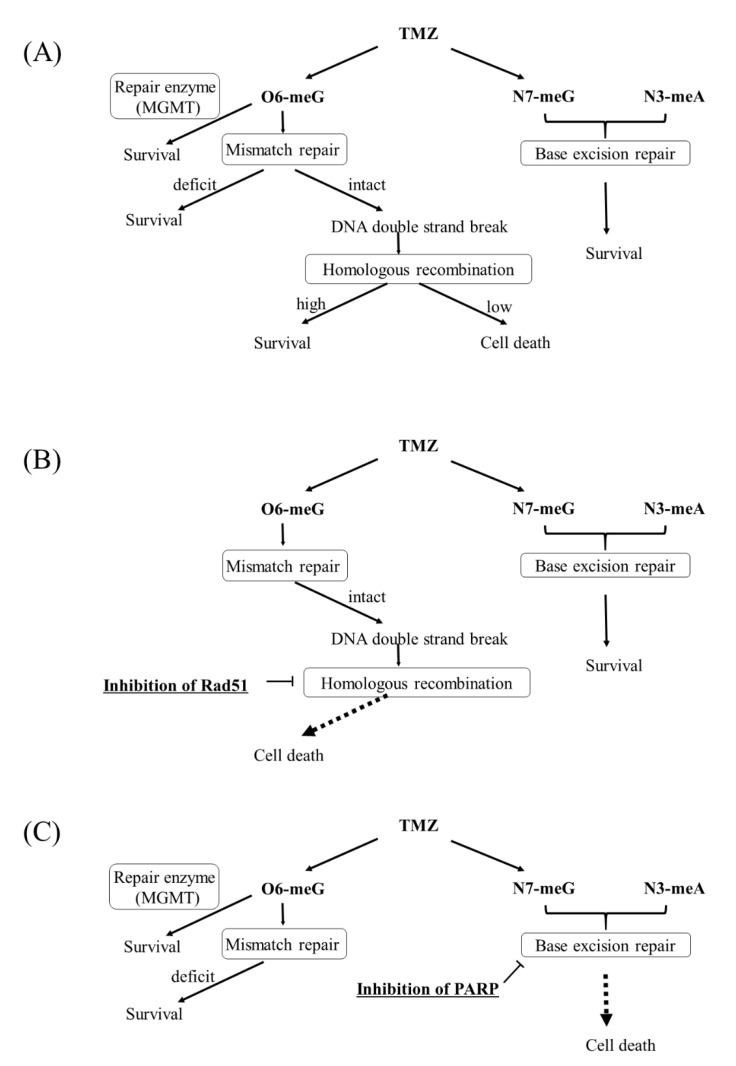
Mechanisms of resistance to temozolomide and methods to overcome the resistance. (**A**) Mechanisms of TMZ-induced DNA damage and mechanism of resistance to the action (**B**) The way to overcome TMZ-resistant cells due to increased homologous recombination. Inhibition of homologous recombination sensitize the cells to TMZ if MGMT is absent and MMR is intact. (**C**) The way to overcome TMZ-resistant cells due to MMR deficiency or high-expressed MGMT. Inhibition of PARP suppresss base excision repair that restore TMZ-induced N7-meG and N3-meA, which leads cell death.

The resistance to TMZ is a serious problem clinically. Because glioblastoma is heterogeneous tumor, the mechanisms of resistance to TMZ have been considered to vary. Corresponding to the heterogeneity of glioblastoma, several monoclonal clones resistant to TMZ were made, and each mechanism of resistance to TMZ was evaluated in the study (Figure 8A). Consistent with clinical reports [15], resistance to TMZ in most clones was associated with deficient MMR. The expression of MGMT was not found in these resistant clones, consistent with previous studies [19,20] although some authors reported that MGMT activity was increased in recurrent cases relative [21].

TMZ-induced DNA DSBs have been reported to be repaired by HR [22]. Because TMZ-induced DNA DSBs and the foci of RAD51 disappeared in parallel, HR was considered to have contributed to the resistance to TMZ in U251TMZR#3 clone. Although there has been no report showing that increased HR was consequent to the resistance in clinical samples of glioblastoma, U251TMZR#3 showed high HR ability compared with parental U251 cells. Importantly, inhibition of HR was found to be useful for resensitizing these types of cells to TMZ (Figure 8B). Development of inhibitors of HR has been promising.

Inhibition of HR did not enhance TMZ-induced cytotoxicity in U251TMZR#8 where MMR-related proteins were suppressed. Previously we showed that none of G2 checkpoint inhibitors resensitized glioma cells with MMR deficiency, suggesting that it was hard to enhance TMZ-induced cytotoxicity in MMR-deficient cells by modifying O6-methylguanine-related pathway [23]. Because TMZ-induced N7-methylguanine and N3-methyladenine were repaired by BER [4] the combination treatment of a BER inhibitor, such as PARP inhibitors and TMZ was found to be effective (Figure 8C). The effect of a PARP inhibitor on enhancement of TMZ-induced cytotoxicity was shown in several resistant clones originated from U251 (p53 mutant) or U87 (p53 wild type), suggesting that the effect was independent of the status of p53. The effect was also found in resistant cells originated from U87 transfected HPV16E6 (U87-E6) [5] (Figure S5), meaning the effect was not related to p53. Because approximately 40% of primary glioblastomas showed p53 mutations [24], the p53-independent effect was very encouraging.

In clinical cases, approximately half of glioblastomas express MGMT at the primary tissues [25], and TMZ is less effective in those cases. The combination treatment of a PARP inhibitor and TMZ was shown to be effective in cells with MGMT expression (Figure 8C). On the basis of these results, the combination treatment of a PARP inhibitor and TMZ appears to be promising for the treatment of glioma harboring several types of resistant mechanisms. However, in a previous study using an orthotopic tumor model, combined treatment of talazoparib with TMZ did not increase survival relative to that of TMZ alone [26]. The researchers explained that lack of the talazoparib effect was due to the limitation of transportation through the BBB. On the other hand, veliparib, which can penetrate the BBB, has been shown to sensitize TMZ in TMZ-sensitive cells in vivo [27,28], but it did not enhance the effect of TMZ on TMZ-resistant cells [27]. Another PARP inhibitor, olaparib, was reported to penetrate the core and margins of recurrent glioblastoma [29]. Several ongoing clinical trials are using these PARP inhibitors, and several biomarkers for good responses have been observed [30,31]. Development of a method to deliver PARP inhibitors to intracranial tumors will contribute to studies on improvement of survival in gliomas. As other mechanisms affecting the response to TMZ, ABCG2 has been reported to be associated with the resistance to TMZ [31,32]. However, the expression of ABCG2 was not changed in our resistant clones compared with parental cells as shown in Figure 1C.

Recently, a new type of DNA alkylating agent, VAL-083, has been used in a clinical trial for treatment of glioblastoma [33]. Importantly, VAL-083 crosses the BBB and preferentially accumulates in tumor tissue [10]. VAL-083 reduced cellular proliferation in a dose-dependent manner in a subcutaneous xenograft tumor model [33]. Our study showed that VAL-083 induced cytotoxicity in cells with several types of resistance mechanisms, which demonstrated that its activity was independent of the status of MGMT, MMR, and p53. This universal effect is expected to lead to good antitumor response for treatment of gliomas.

Finally, we investigated methods to enhance VAL-083-induced cytotoxicity. We showed that VAL-083 induced p-chk1 activation and G2/M arrest. Because the damage induced by VAL-083 was repaired during the G2/M phase, inhibition of chk1 was considered to enhance the cytotoxicity induced by VAL-083. Our data suggested that a chk1 inhibitor suppresses the repair of VAL-083-induced DSBs, which leads to apoptosis in cells.

Because γ-H2AX foci and Rad51 foci were co-localized in the cells treated with VAL-083, VAL-083–induced DNA DSBs were suggested to be repaired by HR, which was consistent with the previous report using lung cancer [34]. Therefore, inhibition of HR was supposed to increase the VAL-083–induced DNA damage. Genetic inhibition of Rad51 indeed heightened the effect of VAL-083 on reduction of cell proliferation. By combined treatment with a chk1 inhibitor or HR inhibitor, the dosage of VAL-083 could be reduced to avoid its adverse effect. Although VAL-083 has been investigated in clinical trials but not yet approved for gliomas, it has been approved for chronic myeloid leukemia and lung cancer in China. After approval, VAL-083 will help to improve the prognosis of recurrent gliomas already treated with TMZ.

## 5. Conclusions

This study demonstrated that several mechanisms were associated with resistance to TMZ even in the same glioma cells, which is similar to the TMZ resistance observed in real patients with glioma. Additionally, the study identified methods for overcoming each mechanism of resistance. Selecting treatments appropriate for the various types of resistance mechanisms potentially could improve the prognosis of patients with gliomas.

## Data Availability

The data presented in this study are available upon request from the corresponding author.

## Supplementary Materials

The following are available online at https://www.mdpi.com/article/10.3390/cancers13112570/s1, Figure S1: The expression of MMR-related proteins in U251 parental and U251-derived TMZ resistant cells. Figure S2: Effect of talazoparib on TMZ-resistant U251 cells treated with TMZ. Figure S3: Effect of talazoparib on TMZ-resistant U87 cells treated with TMZ. Figure S4: Effect of VAL-083 on TMZ-resistant glioma cells. Figure S5: Effect of talazoparib on TMZ-resistant U87-E6 cells treated with TMZ.
