# Supplementary material for: Inhibition of DNA Repair in Combination with Temozolomide or Dianhydrogalactiol Overcomes Temozolomide-Resistant Glioma Cells
Source: Cancers (Basel). 2021 May 24;13(11):2570. doi: 10.3390/cancers13112570 (PMC8197190; doi:10.3390/cancers13112570)
Supplement: Supplementary file 1 [file cancers-13-02570-s001.zip › cancers-1201956-supplementary.pdf]

cancers

Supplementary

Inhibition of Dna Repair in Combination with Temozolomide or Dianhydrogalactiol Overcomes TemozolomiDe-resistant Glioma Cells

Shigeo Ohba *, Kei Yamashiro and Yuichi Hirose

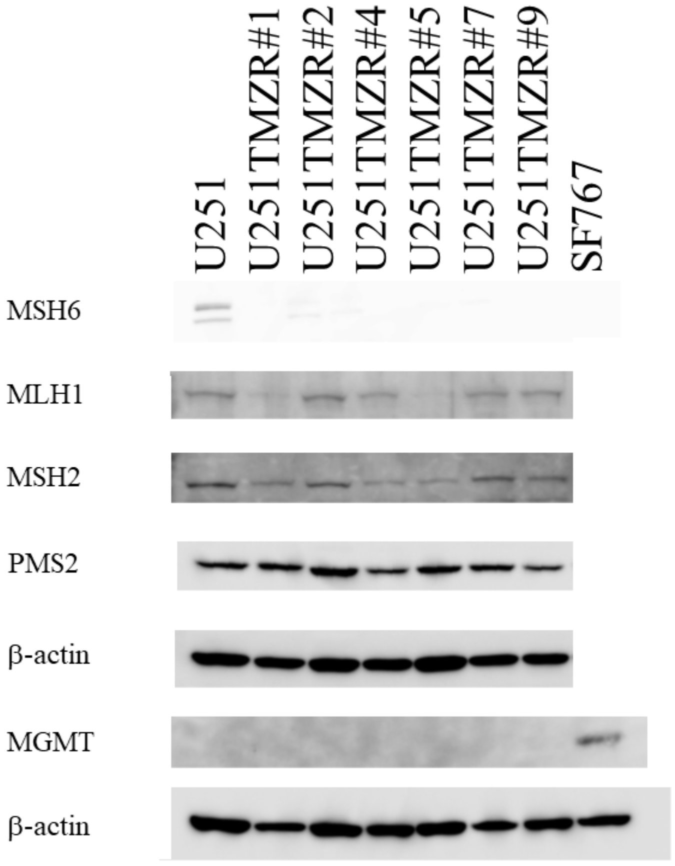

Figure S1. The expression of MMR-related proteins in U251 parental and U251-derived TMZ resistant cells. Western blot analysis of MMR-related proteins (MLH1, MS2H, MSH6, and PMS2), MGMT and β-actin levels in U251 and U251-derived TMZ resistant clones #1, #2, #4, #5, #8, and #9. SF767 cells were used as positive control for MGMT expression.

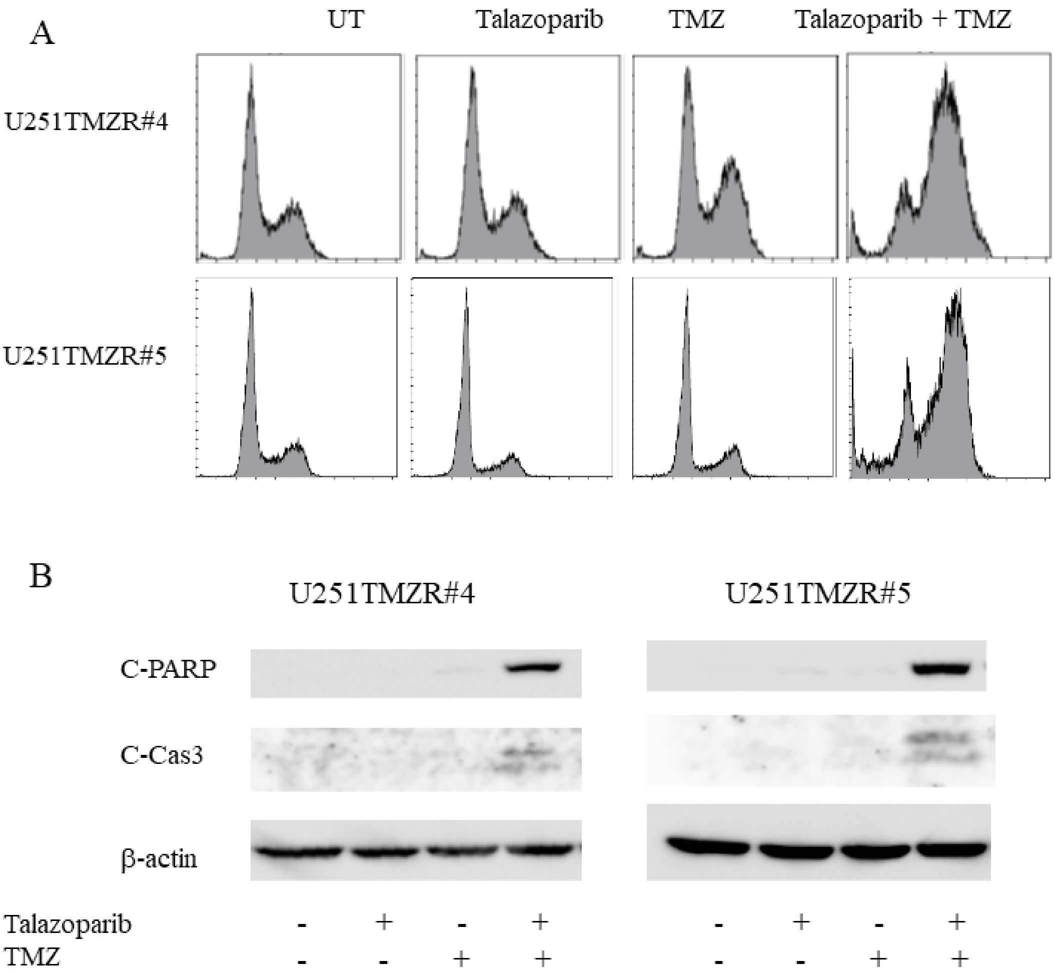

Figure S2. Effect of talazoparib on TMZ-resistant U251 cells treated with TMZ. (**A**) Fluorescence-activated cell sorting analysis of cell cycle distribution in U251-derived TMZ-resistant clone #4 (U251TMZR#4) and #5 (U251TMZR#5) after talazoparib and/or TMZ exposure. (**B**) Western blot analysis of cleaved PARP and β-actin levels in U251TMZR#4 and U251TMZR#5 clones treated with talazoparib and/or TMZ.

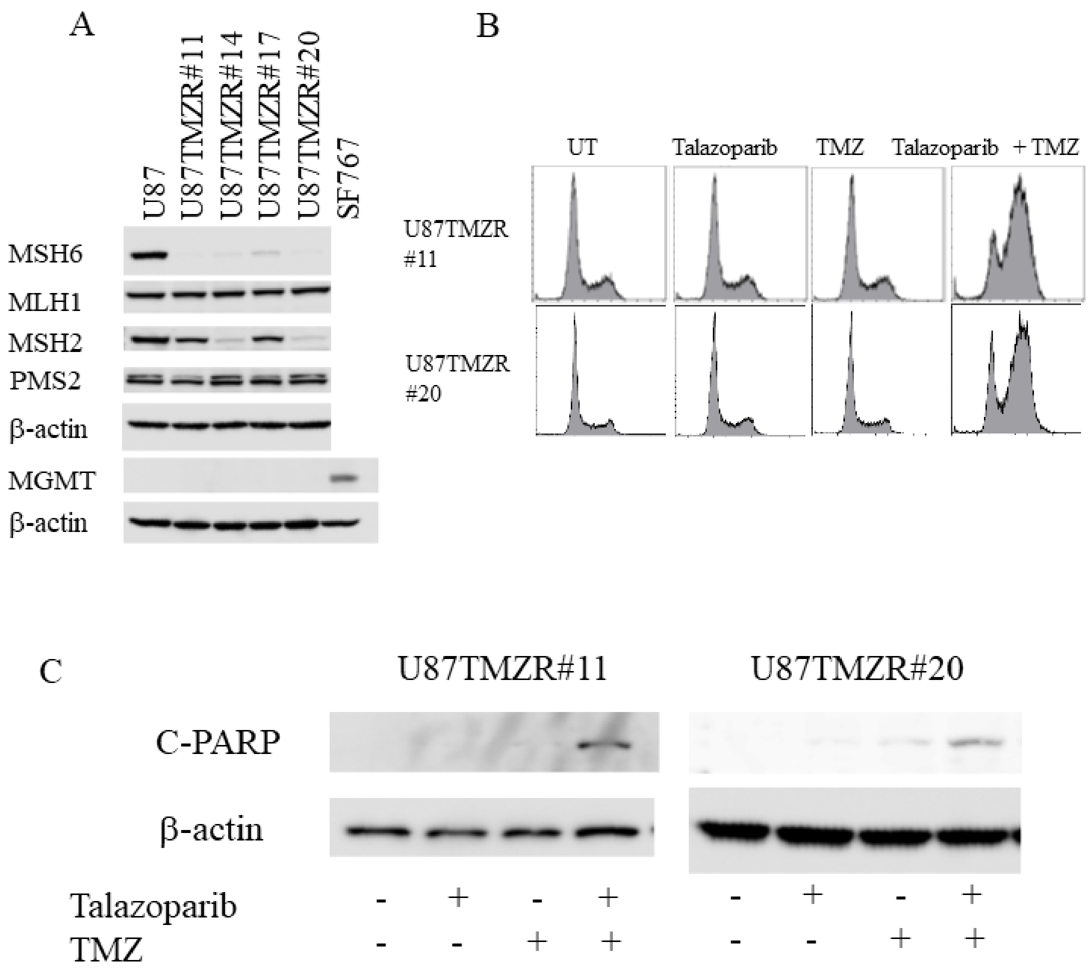

Figure S3. Effect of talazoparib on TMZ-resistant U87 cells treated with TMZ. (**A**) Western blot analysis of MMR-related proteins (MLH1, MS2H, MSH6, and PMS2), MGMT, and β-actin levels in U87 and U87-derived TMZ-resistant clones #11, #14, #17, #20, and SF767. (**B**) Fluorescence-activated cell sorting analysis of cell cycle distribution in U87-derived TMZ resistant clone #11 (U87TMZR#11) and #20 (U87TMZR#20) after talazoparib and/or TMZ exposure. (**C**) Western blot analysis of cleaved PARP and β-actin levels in U87TMZR#11 and U87TMZR#20 clones treated with talazoparib and/or TMZ.

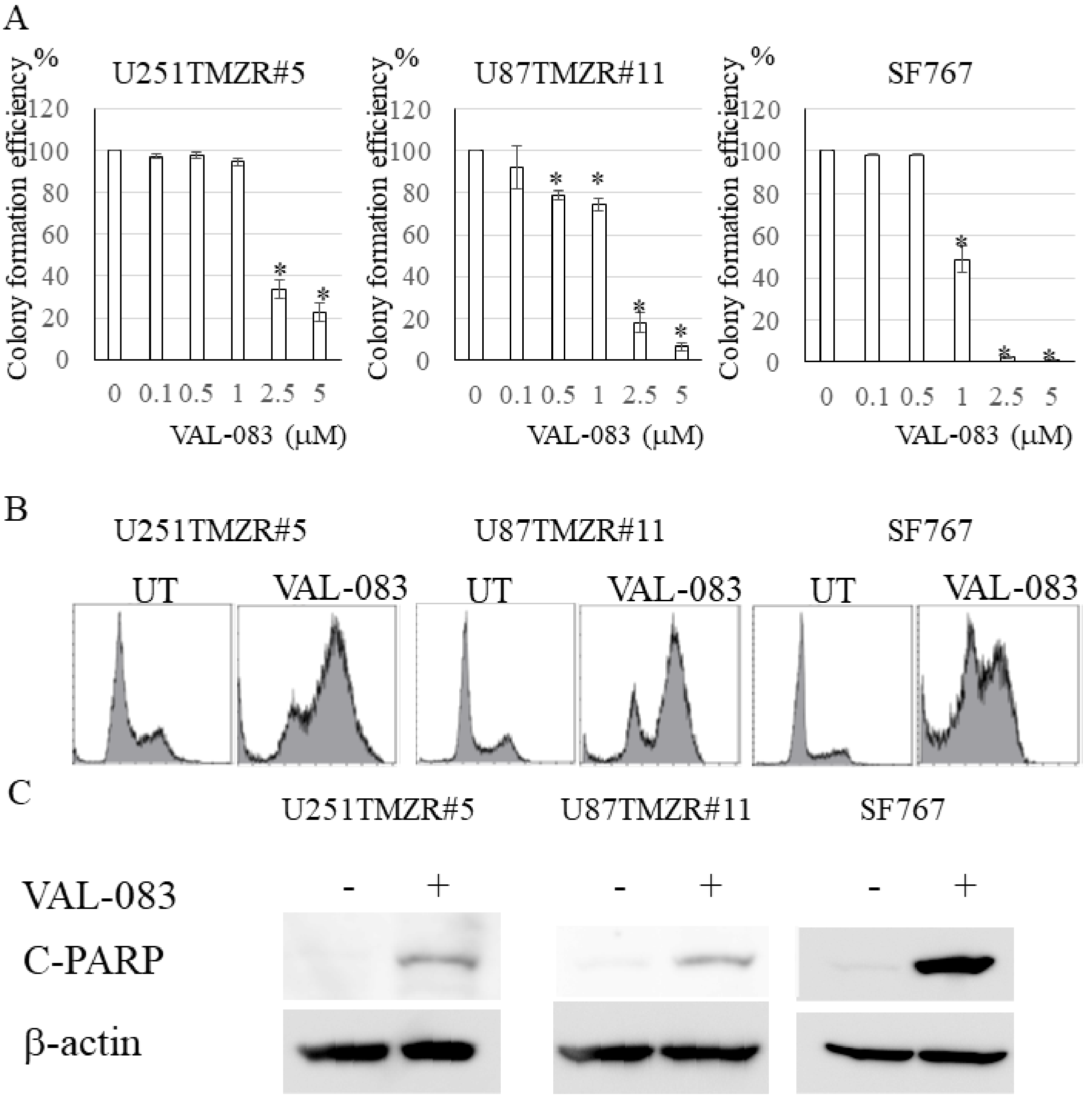

Figure S4. Effect of VAL-083 on TMZ-resistant glioma cells. (**A**) Colony formation efficiency of U251TMZR#5, U87TMZR#11, and SF767 cells following VAL-083 exposure (0–5 mM, 3 days). (**B**) Fluorescence-activated cell sorting analysis of cell cycle distribution in U251TMZR#5, U87TMZR#11, and SF767 cells after VAL-083 exposure. (**C**) Western blot analysis of cleaved PARP and β-actin levels in U251TMZR#5, U87TMZR#11, and SF767 cells treated with VAL-083; *, $p < 0.05$.

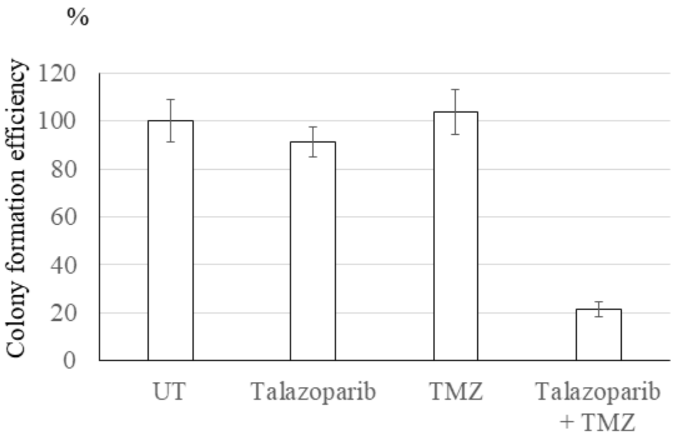

Figure S5. Effect of talazoparib on TMZ-resistant U87-E6 cells treated with TMZ. Colony formation efficiency of TMZ-resistant U87-E6 cells following talazoparib (2.5 nM, 4 days), and /or TMZ (100 μM, 3 h).
